# New pattern in regular nuclei based on their experimental quadrupole transition rates and some new candidates

**DOI:** 10.1038/s41598-023-31002-2

**Published:** 2023-03-08

**Authors:** Asgar Hosseinnezhad, Masoud Seidi, Hadi Sabri

**Affiliations:** 1 Department of Physics, University of Tabriz, P.O. Box 51664-16471, Tabriz, Iran; 2 Department of Physics, University of Ilam, P.O. Box 61391-7711, Ilam, Iran

**Keywords:** Nuclear physics, Theoretical nuclear physics

## Abstract

In this paper, we tried to get a new signature of regular nuclei based on their quadrupole transition rates. We have analyzed the experimental electric quadrupole transition probabilities of well-known "regular nuclei". The results indicate finding specific repetition patterns for E2 transition rates, similar to what has been reported for the energy levels of these nuclei. We also tested the existence of this observed repetition scheme for all known isotopes whose experimental transition rates are available and introduced several new candidates as regular nuclei. Then, the energy spectra (Experimental) of these new suggested "regular nuclei" are investigated in the framework of the Interacting Boson Model, in which the parameters of Hamiltonian confirm the placement of these nuclei in the "Alhassid-Whelan arc of regularity" region. In order to further study the statistical distribution of experimental energy levels related to the electromagnetic transitions we are considering, we studied using the random matrix theory. The results confirmed their regularity.

## Introduction

The statistical investigations on the energy spectra, and transition rates of physical systems that compare their spectral situation with three limits of random matrix theory (RMT) and Poisson distribution, give this opportunity to predict possible correlation of considered data^1–13^. This correlation may regard as the signatures of hidden symmetries or related to the nature of involved forces in considered systems. This correlation yields due to meaningful relationships between the considered samples. Therefore, one would expect that there would be a specific pattern(s) that the systems should follow to show a correlation. Such specific repetition patterns are reported for the energy spectra of nuclear systems corresponding to the three dynamic limits of the interacting boson model (in which the existence of correlation and regularity is doubtless).

In 1991, Alhassid and Whelan studied the chaotic properties of the interacting boson model (IBM) by using the concepts of RMT and introduced an area called the Alhassid-Whelan arc of regularity^2^. They showed that the nuclei in this region (similar to isotopes located in the three limits of IBM) follow a Gaussian orthogonal ensemble (GOE)-like behavior in their spectral statistics. According to the concepts of RMT in nuclear systems, if the statistical distribution of data is consistent with the GOE distribution, the system is regular, and there is a correlation between the data^3,4^. The regularity in this region and the study of observed phenomena related to nuclear structure in this area was the subject of many studies in recent years and received much attention^5,12,14–19^. The works of Amon and Casten are of such studies in which they showed that there is a specific repetition pattern for the energy levels of the nuclei located in the Alhassid-Whelan arc of regularity regular region^5,6^. These authors have introduced several regular nuclei (by examining energy levels). In the Z = 40–100 region, they chose isotopes with 2.2 ≤ R_4/2_ ≤ 3.30 (to select collective nuclei that are not adjacent to the dynamic symmetry U(5) or SU(3)). As a result, several isotopes were introduced as regular nuclei according to the $\left| {R_{\beta \gamma } } \right| = \frac{{\left| {E\left( {0_{\beta }^{ + } } \right) - E\left( {2_{\gamma }^{ + } } \right) } \right|}}{{E\left( {2_{{\text{g}}}^{ + } } \right)}} < 0.025$ condition. R_βγ_ ratio is also studied in Ref.^20^.

The nuclei known as regular nuclei include ^104,106^Pd, ^110,118^Cd, ^120^Xe, ^124,136^Ba, ^136,138^Ce, ^140^Nd, ^144,156, 158^Gd, ^156^Dy, ^156,158^Er, ^170^Yb, ^170,172, 178^Hf, ^176,178^W, and ^178,180^Os. These regular nuclei have been the subject of numerous studies^5,12,14–19^.

**Figure 1:**
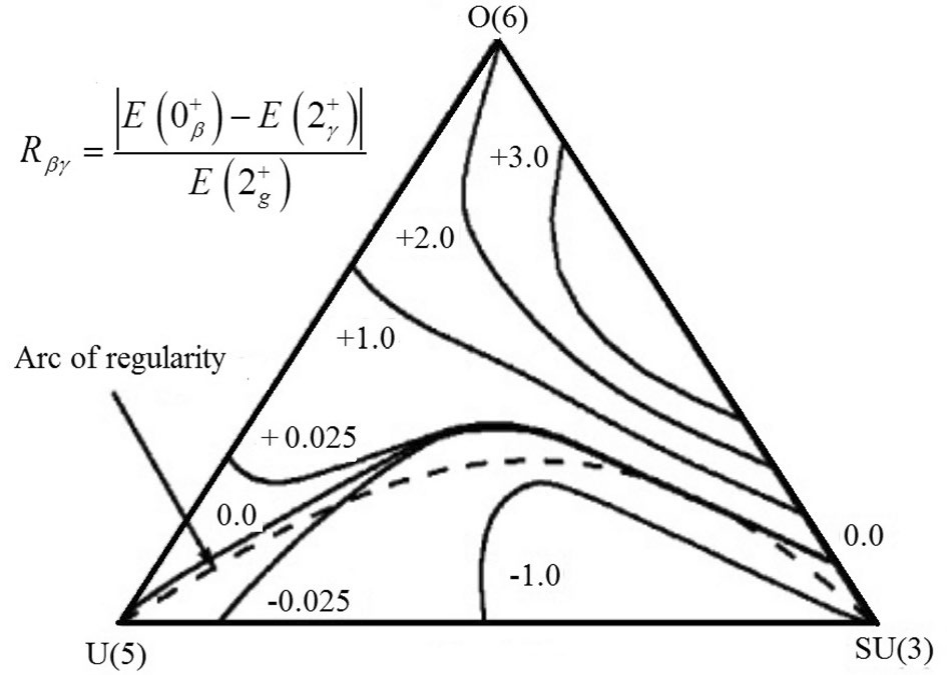
Standard symmetry triangle which is similar with the predictions of Ref.^17^. Different values of $R_{\beta \gamma }$ and also the location of regular nuclei are identified obviously.

IBM is the most common and best choice for describing symmetries in nuclear structures and can describe the collective properties of even-even nuclei. Among the studies that have been done in recent years using IBM to study the nuclear structure, we can mention the Refs.^10,21–30^. In IBM^31^, the dynamic symmetries U(5), O(6), and SU(3) represent spherical, γ-unstable, and deformed nuclei, respectively. This classification is according to the shape and structure of the nuclei, as shown in Fig. 1^32^. These three dynamical limits correspond with the vertices of the triangle. Most known isotopes coincide with the intermediate regions of these vertices. In the extended symmetry triangle, $\overline{{{\text{SU}}\left( 3 \right)}}$ symmetry limit is added to the transition regions^33^.

The statistical investigations on the IBM Hamiltonian (which describes three dynamical limits and transitional regions between these limits) classify systems as regular and chaotic. The regularity originated from pure Hamiltonian includes the Casimir operators of a single dynamical limit without mixing other symmetry chains. Therefore, it corresponds to the correlation of spectral distributions. On the other hand, for such systems that are described by mixed Hamiltonians and located in the transitional regions of three limits, deviation from regularity and a Poisson-like behavior are expected. The work of Alhassid and Whelan^2^ (which has been done based on the results of both classical and quantum chaos) suggests a new regular region called the "Alhassid-Whelan arc of regularity". This arc is connected inside the triangle from the U(5) to the SU(3) limits. This paradox, regularity, and intermediate position between dynamical limits are subjects that L. Amon and R. F. Casten described via a pattern of energy levels.

In this paper, we aim to study the regularity in this region by using the quadrupole transition probability ratios. That means we want to introduce a new observable that repeats similar to the repetition pattern of energy levels but based on the transition probabilities. Therefore, we study using the experimental values of electric quadrupole transition without any effect due to the theoretical models. After confirming the same pattern for the new observable, the next step is to check for these same patterns in other nuclei. Therefore, we aim to introduce new candidates with the same pattern. In the last step, we analyzed the energy spectra of these newly suggested regular nuclei via the IBM Hamiltonian to confirm the location of the new candidates and their placement within the arc of regularity.

## Methods and results

The electromagnetic transition probabilities are sensitive to nuclear structure and measured by improved experimental techniques. Also, electromagnetic transition probabilities are considered commonly used parameters in theoretical predictions. Symmetry mixing and partial dynamical symmetries are new phenomena, as well as are identified by studying these transition rates. Hence, the pattern of these transitions can be used as identification for different nuclei. And one may expect an iteration of them in systems with similar symmetries. On the other hand, the correlation of spectral statistics in "regular nuclei" yields due to the presence of a definite pattern in their energy spectra. In^5,6^, the authors introduced a repetition pattern by using the ratio of energy levels for the well-known regular nuclei located in the region known as the Alhassid-Whelan arc of regularity. The existence of such patterns in the energy spectra of the "regular nuclei"; encourages us to search for possible repetition schemes based on their quadrupole transitions. Also, to eliminate the effects due to the theoretical assumptions of any model in the determination of such observables, we used all the available empirical data^34^ to suggest new patterns in regular nuclei. Similar to Ref.^5^, which introduced different ratios of quadrupole transition rates, we used the following relations for 12 different quadrupole transitions between the levels of ground, gamma and beta bands to examine possible repetitious values. We used the $R_{a,b}^{i,j}$ symbol for these ratios which the superscripts variables define the spin of states which the quadrupole transition are happened between them and the subscript parameters are referred to different energy bands which the considered states belong them. Also, for $4_{\beta }^{ + } \to 2_{g}^{ + }$ and $2_{\gamma }^{ + } \to 2_{g}^{ + }$ quadrupole transitions which are used in two different ratios and compared with two different transitions,$2_{g}^{ + } \to 0_{g}^{ + }$ and $4_{g}^{ + } \to 2_{g}^{ + }$, we used $R_{\beta g}^{{{{4,2} \mathord{\left/ {\vphantom {{4,2} {2,0}}} \right. \kern-0pt} {2,0}}}}$ and $R_{\beta g}^{{{{4,2} \mathord{\left/ {\vphantom {{4,2} {4,2}}} \right. \kern-0pt} {4,2}}}}$ symbols for ratios are correspond with $4_{\beta }^{ + } \to 2_{g}^{ + }$ transitions. Similarly, the $R_{\gamma g}^{{{{2,2} \mathord{\left/ {\vphantom {{2,2} {2,0}}} \right. \kern-0pt} {2,0}}}}$ and $R_{\gamma g}^{{{{2,2} \mathord{\left/ {\vphantom {{2,2} {4,2}}} \right. \kern-0pt} {4,2}}}}$ symbols are used for ratios which related to $2_{\gamma }^{ + } \to 2_{g}^{ + }$ transitions. These ratios are:

1 $$ \begin{gathered} R_{ + g}^{0,2} = \frac{{\left| {{\text{B}}\left( {{\text{E}}2;0_{{\upbeta }}^{ + } \to 2_{{\text{g}}}^{ + } } \right) - {\text{B}}\left( {{\text{E}}2;2_{{\text{g}}}^{ + } \to 0_{{\text{g}}}^{ + } } \right){ }} \right|}}{{{\text{B}}\left( {{\text{E}}2;2_{{\text{g}}}^{ + } \to 0_{{\text{g}}}^{ + } } \right)}} \hfill \\ R_{\gamma g}^{2,0} = \frac{{\left| {{\text{B}}\left( {{\text{E}}2;2_{{\upgamma }}^{ + } \to 0_{g}^{ + } } \right) - {\text{B}}\left( {{\text{E}}2;2_{{\text{g}}}^{ + } \to 0_{{\text{g}}}^{ + } } \right){ }} \right|}}{{{\text{B}}\left( {{\text{E}}2;2_{{\text{g}}}^{ + } \to 0_{{\text{g}}}^{ + } } \right)}} \hfill \\ R_{\beta g}^{4,2/2,0} = \frac{{\left| {{\text{B}}\left( {{\text{E}}2;4_{{\upbeta }}^{ + } \to 2_{{\text{g}}}^{ + } } \right) - {\text{B}}\left( {{\text{E}}2;2_{{\text{g}}}^{ + } \to 0_{{\text{g}}}^{ + } } \right){ }} \right|}}{{{\text{B}}\left( {{\text{E}}2;2_{{\text{g}}}^{ + } \to 0_{{\text{g}}}^{ + } } \right)}} \hfill \\ R_{\beta g}^{2,0} = \frac{{\left| {{\text{B}}\left( {{\text{E}}2;2_{{\upbeta }}^{ + } \to 0_{{\text{g}}}^{ + } } \right) - {\text{B}}\left( {{\text{E}}2;2_{{\text{g}}}^{ + } \to 0_{{\text{g}}}^{ + } } \right){ }} \right|}}{{{\text{B}}\left( {{\text{E}}2;2_{{\text{g}}}^{ + } \to 0_{{\text{g}}}^{ + } } \right)}} \hfill \\ R_{\beta g}^{4,2/4,2} = \frac{{\left| {{\text{B}}\left( {{\text{E}}2;4_{{\upbeta }}^{ + } \to 2_{{\text{g}}}^{ + } } \right) - {\text{B}}\left( {{\text{E}}2;4_{{\text{g}}}^{ + } \to 2_{{\text{g}}}^{ + } } \right){ }} \right|}}{{{\text{B}}\left( {{\text{E}}2;4_{{\text{g}}}^{ + } \to 2_{{\text{g}}}^{ + } } \right)}} \hfill \\ R_{\beta g}^{4,4} = \frac{{\left| {{\text{B}}\left( {{\text{E}}2;4_{{\upbeta }}^{ + } \to 4_{{\text{g}}}^{ + } } \right) - {\text{B}}\left( {{\text{E}}2;4_{{\text{g}}}^{ + } \to 2_{{\text{g}}}^{ + } } \right){ }} \right|}}{{{\text{B}}\left( {{\text{E}}2;4_{{\text{g}}}^{ + } \to 2_{{\text{g}}}^{ + } } \right)}} \hfill \\ R_{\gamma g}^{2,2/4,2} = \frac{{\left| {{\text{B}}\left( {{\text{E}}2;2_{{\upgamma }}^{ + } \to 2_{{\text{g}}}^{ + } } \right) - {\text{B}}\left( {{\text{E}}2;4_{{\text{g}}}^{ + } \to 2_{{\text{g}}}^{ + } } \right){ }} \right|}}{{{\text{B}}\left( {{\text{E}}2;4_{{\text{g}}}^{ + } \to 2_{{\text{g}}}^{ + } } \right)}} \hfill \\ R_{\gamma g}^{2,4} = \frac{{\left| {{\text{B}}\left( {{\text{E}}2;2_{{\upgamma }}^{ + } \to 4_{{\text{g}}}^{ + } } \right) - {\text{B}}\left( {{\text{E}}2;2_{{\text{g}}}^{ + } \to 0_{{\text{g}}}^{ + } } \right){ }} \right|}}{{{\text{B}}\left( {{\text{E}}2;2_{{\text{g}}}^{ + } \to 0_{{\text{g}}}^{ + } } \right)}} \hfill \\ R_{\gamma g}^{2,2/2,0} = \frac{{\left| {{\text{B}}\left( {{\text{E}}2;2_{{\upgamma }}^{ + } \to 2_{{\text{g}}}^{ + } } \right) - {\text{B}}\left( {{\text{E}}2;2_{{\text{g}}}^{ + } \to 0_{{\text{g}}}^{ + } } \right){ }} \right|}}{{{\text{B}}\left( {{\text{E}}2;2_{{\text{g}}}^{ + } \to 0_{{\text{g}}}^{ + } } \right)}} \hfill \\ R_{\gamma g}^{4,2} = \frac{{\left| {{\text{B}}\left( {{\text{E}}2;4_{{\upgamma }}^{ + } \to 2_{{\text{g}}}^{ + } } \right) - {\text{B}}\left( {{\text{E}}2;4_{{\text{g}}}^{ + } \to 2_{{\text{g}}}^{ + } } \right){ }} \right|}}{{{\text{B}}\left( {{\text{E}}2;4_{{\text{g}}}^{ + } \to 2_{{\text{g}}}^{ + } } \right)}} \hfill \\ R_{\gamma g}^{4,4} = \frac{{\left| {{\text{B}}\left( {{\text{E}}2;4_{{\upgamma }}^{ + } \to 4_{{\text{g}}}^{ + } } \right) - {\text{B}}\left( {{\text{E}}2;4_{{\text{g}}}^{ + } \to 2_{{\text{g}}}^{ + } } \right){ }} \right|}}{{{\text{B}}\left( {{\text{E}}2;4_{{\text{g}}}^{ + } \to 2_{{\text{g}}}^{ + } } \right)}} \hfill \\ R_{\beta g}^{4,6} = \frac{{\left| {{\text{B}}\left( {{\text{E}}2;4_{{\upbeta }}^{ + } \to 6_{{\text{g}}}^{ + } } \right) - {\text{B}}\left( {{\text{E}}2;6_{{\text{g}}}^{ + } \to 4_{{\text{g}}}^{ + } } \right){ }} \right|}}{{{\text{B}}\left( {{\text{E}}2;6_{{\text{g}}}^{ + } \to 4_{{\text{g}}}^{ + } } \right)}} \hfill \\ \end{gathered} $$

The results of the calculations by using experimental values are shown in Table 1. We used the explicit values of these transitions, which are listed in different data sheets which are available in Ref^34^ independent of the experimental method and the measurement errors. The results are not reported for the ^118^Cd, ^120^Xe, ^124^Ba, ^136,138^Ce, ^140^Nd, ^144^Gd, ^156,158^Er, ^170,172^Hf, ^176,178^W, and ^178,180^Os nuclei in Table 1. The reason is that there is no experimental value corresponding to any of the ratios R_1_ to R_12_ for these nuclei. Also, in Tables 1 and 2, there are no experimental data related to some ratios, which we indicate with a "-" symbol.

**Table 1:** The ratio of quadrupole transition rates in regular nuclei.

Regular nuclei	$R_{{{\upbeta }g}}^{0,2}$	$R_{\gamma g}^{2,0}$	$R_{\beta g}^{4,2/2,0}$	$R_{\beta g}^{2,0}$	$R_{\beta g}^{4,2/4,2}$	$R_{\beta g}^{4,4}$	$R_{\gamma g}^{2,2/4,2}$	$R_{\gamma g}^{2,4}$	$R_{\gamma g}^{2,2/2,0}$	$R_{\gamma g}^{4,2}$	$R_{\gamma g}^{4,4}$	$R_{\beta g}^{4,6}$
${}_{46}^{104} {\text{Pd}}$	0.64	0.99	0.97	–	0.98	–	–	–	–	–	–	–
${}_{46}^{106} {\text{Pd}}$	0.21	0.99	–	0.97	–	–	–	0.93	–	–	–	–
${}_{48}^{110} {\text{Cd}}$	–	0.98	–	0.95	–	–	0.28	–	0.97	0.87	0.71	–
${}_{56}^{136} {\text{Ba}}$	–	0.99	–	0.96	–	–	–	–	–	–	–	–
${}_{64}^{156} {\text{Gd}}$	0.95	0.97	0.99	0.99	0.99	–	0.97	0.99	0.96	0.99	0.96	0.99
${}_{64}^{158} {\text{Gd}}$.	0.99	0.99	0.99	0.98	–	0.99	0.97	0.99	0.96	0.99	0.97	–
${}_{66}^{156} {\text{Dy}}$	–	0.95	0.99	–	0.99	0.94	0.96	0.94	0.93	–	–	0.95
${}_{70}^{170} {\text{Yb}}$	–	0.98	–	0.99	–	–	–	–	0.97	–	–	–
${}_{72}^{178} {\text{Hf}}$	–	0.97	0.99	0.99	0.99	–	0.97	0.99	0.97	–	–	0.99

**Table 2:** E2 transition rate ratios for new candidates for regular nuclei.

Nuclei	${R}_{\beta g}^{\mathrm{4,6}}$	${R}_{\gamma g}^{\mathrm{4,4}}$	${R}_{\gamma g}^{\mathrm{4,2}}$	${R}_{\gamma g}^{\mathrm{2,2}/\mathrm{2,0}}$	${R}_{\gamma g}^{\mathrm{2,4}}$	${R}_{\gamma g}^{\mathrm{2,2}/\mathrm{4,2}}$	${R}_{\beta g}^{\mathrm{4,4}}$	${R}_{\beta g}^{\mathrm{4,2}/\mathrm{4,2}}$	${R}_{\beta g}^{\mathrm{2,0}}$	${R}_{\beta g}^{\mathrm{4,2}/\mathrm{2,0}}$	${R}_{\gamma g}^{\mathrm{2,0}}$	${R}_{\upbeta g}^{\mathrm{0,2}}$
${}_{62}{}^{152}\mathrm{Sm}$	–	0.97	–	–	–	–	0.95	0.99	0.93	0.99	0.97	0.99
${}_{62}{}^{154}\mathrm{Sm}$	0.93	0.98	–	0.99	–	–		0.99	–	–	–	–
${}_{64}{}^{154}\mathrm{Gd}$	0.66	0.96	–	0.99	–	–	0.94	0.98	0.92	–	–	–
${}_{66}{}^{158}\mathrm{Dy}$	–	0.96	–	0.98	–	–	0.92	0.98	0.89	–	–	–
${}_{66}{}^{160}\mathrm{Dy}$	–	0.97	–	0.99	–	–	–	0.99	0.95	–	–	–
${}_{64}{}^{160}\mathrm{Gd}$	–	0.98	–	–	–	–	–	0.99	0.96	–	–	–
${}_{68}{}^{162}\mathrm{Er}$	–	0.96	–	0.99	–	–	–	0.99	0.92	–	–	–
${}_{68}{}^{164}\mathrm{Er}$	–	0.97	–	–	–	–	0.95	0.99	0.94	–	–	–
${}_{68}{}^{166}\mathrm{Er}$	0.98	0.97	–	0.99	–	–	0.96	0.99	0.95	0.99	0.96	–
${}_{70}{}^{168}\mathrm{Yb}$	–	0.97	–	0.99	–	–	–	0.99	0.95	–	–	–
${}_{68}{}^{168}\mathrm{Er}$	0.99	0.97	0.99	0.99	0.99	0.99	0.97	0.99	0.96	0.99	0.96	0.98
${}_{68}{}^{170}\mathrm{Er}$	–	0.98	–	0.99	–	–	–	0.99	0.97	–	–	–
${}_{70}{}^{172}\mathrm{Yb}$	0.98	0.99	–	0.99	–	–	–	0.99	–	0.97	0.95	–
${}_{72}{}^{174}\mathrm{Hf}$	–	0.96	–	0.98	–	–	–	–	0.95	–	–	–
${}_{72}{}^{176}\mathrm{Hf}$	–	0.97	–	0.99	–	–	–	–	–	–	–	–
${}_{72}{}^{180}\mathrm{Hf}$	–	0.97	–	–	–	–	0.97	–	0.96	–	–	–
${}_{74}{}^{182}\mathrm{W}$	–	0.97	–	–	–	–	0.96	0.99	0.95	0.98	0.94	–
${}_{74}{}^{184}\mathrm{W}$	–	0.97	–	0.99	–	–	0.95	0.99	0.93	0.99	0.95	–
${}_{74}{}^{186}\mathrm{W}$	–	0.95	–	–	–	–	0.92	–	0.9	–	–	–
${}_{76}{}^{186}\mathrm{Os}$	–	0.89	–	–	–	–	0.82	0.98	0.74	0.97	0.81	–
${}_{90}{}^{230}\mathrm{Th}$	–	0.98	–	0.98	–	–	0.97	0.99	0.97	–	–	–
${}_{90}{}^{232}\mathrm{Th}$	–	0.98	–	0.98	–	–	0.99	0.99	0.99	–	–	–
${}_{92}{}^{234}\mathrm{U}$	0.99	0.98	–	0.99	–	–	–	0.99	0.99	–	–	–
${}_{92}{}^{238}\mathrm{U}$	–	0.98	–	0.98	–	–	–	0.99	0.98	–	–	–
${}_{94}{}^{238}\mathrm{Pu}$	–	0.98	–	–	–	–	–	0.98	–	–	–	–

The results show that defined ratios are in a determined range (${R}_{a,b}^{i,j}\le 0.99$). Therefore, other data can be easily calculated by having given data (in Eq. 1). These results confirm our idea of the existence of a repetition scheme in transitional probabilities. Also, we considered the possibility to extend the proposed pattern for the electric quadrupole transitions which originated from high-spin levels. The lack of enough experimental data for such transitions in the considered nuclei, make it impossible and therefore, we do not include them.

In Table 1, for ^106^Pd in column 2 and ^110^Cd in column 7, the defined ratio is lower compared to other values. In other words, for these nuclei, we do not see the same pattern as the other nuclei (in the two mentioned ratios). So, they do not have regular behavior for the ${R}_{\upbeta g}^{\mathrm{0,2}}$ ratio of ^106^Pd and for the ${R}_{\gamma g}^{\mathrm{2,2}/\mathrm{4,2}}$ ratio of ^110^Cd.

In the next step, we check this scheme for all nuclei whose experimental transition rates are available^34^. We try to control the possibility of this pattern for other nuclear systems and suggest new candidates for regular nuclei. If this pattern is confirmed for other systems, we must consider the placement of these new candidates in the Casten triangle and "Alhassid-Whelan arc of regularity" region. The results of this test are listed in Table 2.

The results show that the suggested ratios of transition probabilities have values within a certain range ($R_{a,b}^{i,j} \le 0.99$) for the listed nuclei in Table 2. This allows us to propose these nuclei as new candidates for regular nuclei. Now, we consider their location in the Casten triangle. To do this, we must first solve the IBM Hamiltonian and obtain its control parameters. Then, by obtaining the control parameters of IBM Hamiltonian, e.g. *η* & *χ*, we can identify the possible placement of new candidates in the range of the Alhassid-Whelan arc of regularity. As expressed in Refs^18–20^, *η* = 0 to 1 and *χ* = 0 to $- \sqrt {7/2}$ define this arc.

The general Hamiltonian of IBM, which is parameterized in the self-consistent Q formalism, is as follows^35^:

2 $$ H = E_{0} + c_{0} \hat{n}_{d} + c_{1} Q^{\chi } .Q^{\chi } + c_{2} L^{2} $$

$\hat{n}_{d} = d^{\dag } .d$ represents the number of d bosons, *L* represents the angular momentum, and *Q*^*χ*^ represents the quadrupole operator as follows:

3 $$ Q^{\chi } = \left[ {d^{\dag } \times \tilde{s} + s^{\dag } \times \tilde{d}} \right]^{\left( 2 \right)} + \chi \left( {d^{\dag } \times d{ }} \right)^{\left( 2 \right)} $$

*χ* is a control parameter. The *c*_0_ = 1 value represents the vibrational nuclei (that correspond with U(5) dynamical limit). The *c*_0_ = 0 and $\chi = { } - \sqrt {7/2}$ requirements are used to describe the rotational nuclei (SU(3) dynamical limit). Also, *c*_0_ = 0 and *χ* = 0 correspond with the O(6) dynamical limit and describe the γ-unstable nuclei.

We have followed the original method introduced by Alhassid and Whelan (in the identification of the arc of regularity). They used the classical Hamiltonian of IBM and by fitting the theoretical predictions for the energy spectra and the experimental counterparts, determined the control parameters of Hamiltonian. The classical Hamiltonian is defined as *h*(*α*, α^*^)$\equiv \alpha {|}H{|}\alpha /N$. That *α* and iα^*^ are in the role of canonical conjugate variables^2,36,37^. Equation *h*(*α*, α^*^) is as follows:

4 $$ h\left( {\alpha , \alpha^{*} } \right) = \in_{0} + \overline{c}\left[ {\eta n_{d} - \left( {1 - \eta } \right)\left( {q^{\chi } .q^{\chi } } \right)} \right] + \overline{c}_{1} l^{2} $$

The values of c-functions *n*_*d*_, *q*^*χ*^, and *l* are obtained by dividing the expected values of operators $\hat{n}_{d}$, *Q*^*χ*^, and *L* by the boson number N. Also, the relationship between the parameters of Eqs. (3) and (5) are:

5 $$ \in_{0} =\, \frac{{E_{0} }}{N}, \overline{c}_{1} = Nc_{1} , \overline{c} = \frac{{c_{0} }}{\eta }, \frac{\eta }{1 - \eta } = - \frac{{c_{0} }}{{Nc_{2} }} $$

We used the least-square fit in MATLAB software to extract the control parameters of Eq. (4),* η*, and *χ* in comparison with the available experimental data^34^ for the 0^+^, 2^+^, 4^+^, 6^+^, and 8^+^ energy levels of ground, beta, and gamma bands of the new suggested candidates of the regular nuclei. These control parameters are shown in Table 3. Also, we presented the root mean square (RMS) values as the last column in Table 3 to describe the quality of extraction process for these control parameters. RMS is a good criterion for the applicability of any model which is defined as^20^:

6 $$ \sigma =\, \sqrt {\frac{{\sum\nolimits_{i = 1}^{n} {\left( {E_{i} \left( {\exp } \right) - E_{i} \left( {th} \right)} \right)^{2} } }}{{\left( {n - 1} \right)E\left( {2_{1}^{ + } } \right)}}} $$

**Table 3:** Control parameter values for the regular nuclei candidates.* σ* describes the quality of extraction procedure.

Nuclei	N	η	χ	σ
${}_{62}^{152} {\text{Sm}}$	10	0.64762	− 9.3806	1.23
${}_{62}^{154} {\text{Sm}}$	11	0.23478	− 0.37131	1.10
${}_{64}^{154} {\text{Gd}}$	11	0.60198	− 0.37131	1.09
${}_{66}^{158} {\text{Dy}}$	13	0.85303	− 0.34046	0.96
${}_{66}^{160} {\text{Dy}}$	14	0.9390	− 0.32766	0.71
${}_{64}^{160} {\text{Gd}}$	14	0.97975	− 0.32765	0.95
${}_{68}^{162} {\text{Er}}$	13	0.41727	− 0.34045	0.92
${}_{68}^{164} {\text{Er}}$	14	0.96309	− 0.32765	0.94
${}_{68}^{166} {\text{Er}}$	15	0.90631	− 0.3162	0.47
${}_{70}^{168} {\text{Yb}}$	14	0.90372	− 0.32765	0.56
${}_{68}^{168} {\text{Er}}$	16	0.78052	− 0.30588	0.92
${}_{68}^{170} {\text{Er}}$	17	0.77016	− 0.54277	0.97
${}_{70}^{172} {\text{Yb}}$	16	0.91599	− 0.30588	1.12
${}_{72}^{174} {\text{Hf}}$	15	0.69989	− 0.82885	1.36
${}_{72}^{176} {\text{Hf}}$	16	0.64475	− 0.30588	1.14
${}_{72}^{180} {\text{Hf}}$	14	0.98266	− 0.32766	0.77
${}_{74}^{182} {\text{W}}$	13	0.54701	− 0.34045	1.20
${}_{74}^{184} {\text{W}}$	12	0.43141	− 0.3555	0.94
${}_{74}^{186} {\text{W}}$	11	0.45054	− 0.37131	1.18
${}_{76}^{186} {\text{Os}}$	11	0.78025	− 0.37131	0.92
${}_{90}^{230} {\text{Th}}$	11	0.86869	− 0.37131	0.79
${}_{90}^{232} {\text{Th}}$	12	0.48925	− 0.35503	0.98
${}_{92}^{234} {\text{U}}$	13	0.92939	− 0.34046	0.54
${}_{92}^{238} {\text{U}}$	15	0.96865	− 0.3162	0.88
${}_{94}^{238} {\text{Pu}}$	15	0.67612	− 0.31621	0.86

The *σ* values show the accuracy of fitting process and consequently, we can conclude about the location of these new suggested candidates with high precision. Our idea for the location of these new candidates (in the arc of regularity) is confirmed by Table 3 results. There is an exception only for ^152^Sm. This confirmation allows us to introduce these candidates as the new regular nuclei. Also, in this table and for some nuclei, ^152-154^Sm, ^154^Gd, ^172^Yb, ^174-176^Hf and ^176&182^W, the RMS values have values more than 1. Such kind of variation may relate to the inadequacy of theoretical treatment. The majority of these nuclei are deformed one and other techniques such as quasi dynamical symmetry (QDS) and partial dynamical symmetry (PDS) may reduce such variations. To this aim, the two-body SU(3)-PDS Hamiltonian in IBM-model are used as:

7 $$ \begin{gathered} \hat{H}_{PDS} = \overset{\lower0.5em\hbox{$\smash{\scriptscriptstyle\frown}$}}{H} \left( {h_{0} ,h_{2} } \right) + \hat{C}(O(3)) = \hat{H}_{DS} + \left( {h_{0} - h_{2} } \right)P_{0}^{\dag } P_{0} \hfill \\ \quad \quad \quad = h_{0} P_{0}^{\dag } P_{0} + h_{2} P_{2}^{\dag } .\tilde{P}_{2} + \hat{C}(O(3)) \hfill \\ \end{gathered} $$

$$P_{0}^{\dag } = d^{\dag } .d^{\dag } - 2\left( {s^{\dag } } \right)^{2}$$ and $$P_{2\mu }^{\dag } = 2d_{\mu }^{\dag } s^{\dag } - \sqrt 7 \left( {d^{\dag } d^{\dag } } \right)_{\mu }^{(2)}$$ describe the boson pair operator with L = 0 and 2 angular momentum, respectively. Also, the *h*_0_& *h*_2_ coefficients describe their effects. For the *h*_0_ = *h*_2_ requirement, the Hamiltonian involves the Casimir operators of U(6) ⊃ SU(3) ⊃ SO(3) chain, hence exhibits an SU(3) dynamical symmetry (DS). Also, for *h*_0_ ≠ *h*_2_, the SU(3) symmetry is broken. In the *h*_0_ = *h*_2_ case, $$\overset{\lower0.5em\hbox{$\smash{\scriptscriptstyle\frown}$}}{H} \left( {h_{0} ,h_{2} } \right)$$ is equal with an SU(3) scalar and for *h*_0_ = -5*h*_2_ case, $$\overset{\lower0.5em\hbox{$\smash{\scriptscriptstyle\frown}$}}{H} \left( {h_{0} ,h_{2} } \right)$$ transforms as $$\left( {\lambda ,\mu } \right)$$ = (2,2), SU(3) tensor component. The solvable states of ground and gamma bands and consequently, the energy spectra of the different levels in these bands introduced by Leviatan as following^24^:

8a $$ \left[ {g,K = 0} \right] \, , \, \left| {N,\left( {2N,0} \right),K = 0,L} \right\rangle { , } \quad E_{PDS} = CL\left( {L + 1} \right) \, , \, L = 0,2,..., \, 2N $$

8b $$ \begin{gathered} \, \left[ {\gamma^{K} ,K = 2k} \right] \, , \, \left| {N,\left( {2N - 4k,2k} \right),K = 2k,L} \right\rangle \, , \, \hfill \\ E_{PDS} = 6h_{2} k\left( {2N - 2k + 1} \right) + DL\left( {L + 1} \right) \, , \, L = K,K + 1,...,(2N - 2k) \hfill \\ \end{gathered} $$

the final form of energy spectra in this approach for the beta band are defined as:

8c $$ E_{\beta } = 4N\left( {2h_{0} + h_{2} } \right) + DL\left( {L + 1} \right){\text{, For large }}N $$

The two parameters, D and *h*_2,_ are determined compared to experimental data, also, the *h*_0_ parameter was varied so as to reproduce the band-head energy of the *β* band. The values of the Hamiltonian parameters derived microscopically from various EDFs, are given in^24^. Bu using this method, the RMS values are obtained as 0.88, 0.91, 0.90, 0.86, 0.81, 0.88, 0.92 and 0.94 for respectively, ^152-154^Sm, ^154^Gd, ^172^Yb, ^174-176^Hf and ^176&182^W which show notable reduction to the reported RMS values in the Table 3 for these nuclei. These results confirm our idea about the advantages of PDS in comparison with DS in description of energy levels in such nuclei. Another subject that we will address in further studies is the structure of wavefunctions for different states of rotational bands (especially the beta band that has specific effects on the transition rates).

The presence of these new candidates in the "arc of regularity" region and therefore, classify as the regular nuclei, may relate to their deformation. Most of well-known regular nuclei which introduced by Amoan and Casten in Refs^5,6^ and the new ones which suggested in this study, are deformed nuclei and their experimental quadrupole deformation satisfy $\beta_{2}^{Exp.} \ge 0.200$. As have reported in different studies such as Refs.^24,42,46^, the spectral statistics of such deformed nuclei must obey the prediction of GOE limit of RMT. Therefore, to further ensure the regularity of these new candidates, we study the statistical behavior of the corresponding levels using RMT. In the mentioned nuclei, the $2_{{\text{g}}}^{ + }$, $4_{{\text{g}}}^{ + }$, $0_{{\upbeta }}^{ + }$, $2_{{\upbeta }}^{ + }$, $4_{{\upbeta }}^{ + }$, $2_{{\upgamma }}^{ + }$, and $4_{{\upgamma }}^{ + }$ energy levels are involved in the studied electromagnetic transitions (as initial and final states) in this paper. So we extract the energy values related to these levels from Ref^34^. Then, using RMT, we examine their regularity in our desired nuclei. For the statistical study, we first unfold the data. To this aim, we tidy the energy level values from the smallest to the largest order. By using the concept of nearest neighbor spacing distribution (NNSD), we must first calculate the following quantity^38–41^:

9 $$ S_{i} = E_{i + 1} - E_{i} $$

Then we calculate the average spacing between the energy levels from equation

10 $$ D = \frac{{\sum S_{i} }}{N} $$

Finally, the unfolded data is obtained from relation

11 $$ s = \frac{{S_{i} }}{D} $$

and we can use them for statistical study.

**Figure 2:**
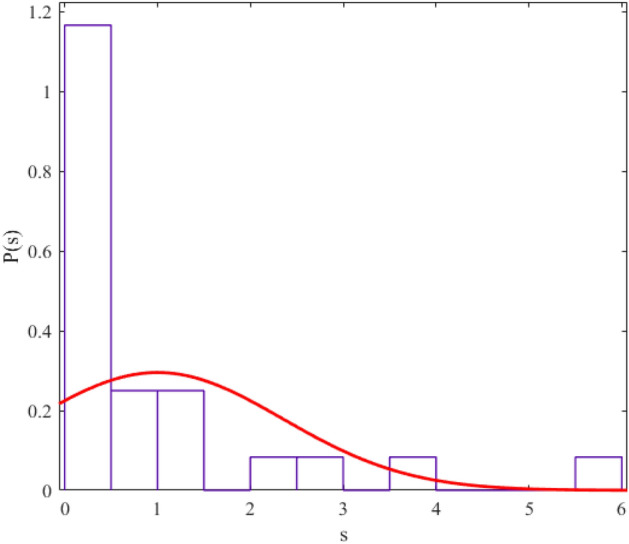
The statistical distribution of $2_{{\text{g}}}^{ + }$ energy level. The histogram presents the unfolded energy levels and the curve describes the Berry-Robnik distribution, Eq. (12).

**Figure 3:**
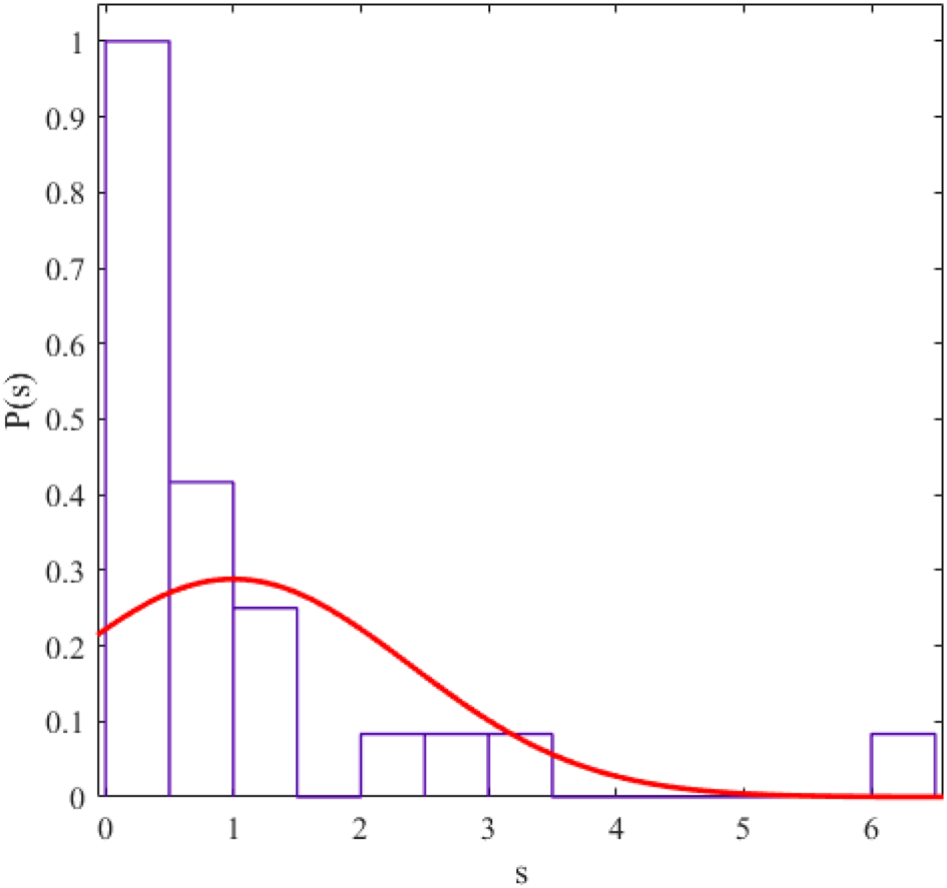
Similar to Fig. 1, the statistical distribution of $4_{{\text{g}}}^{ + }$ energy level.

**Figure 4:**
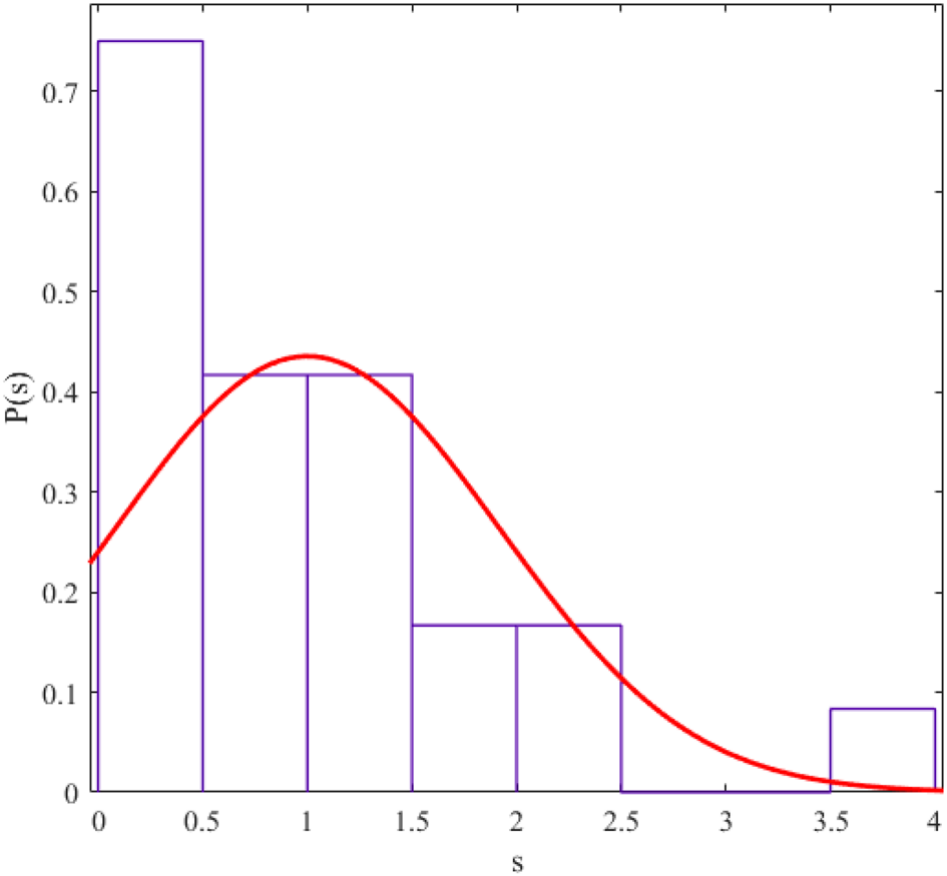
Similar to Fig. 1, the statistical distribution of $0_{\beta }^{ + }$ energy level.

**Figure 5:**
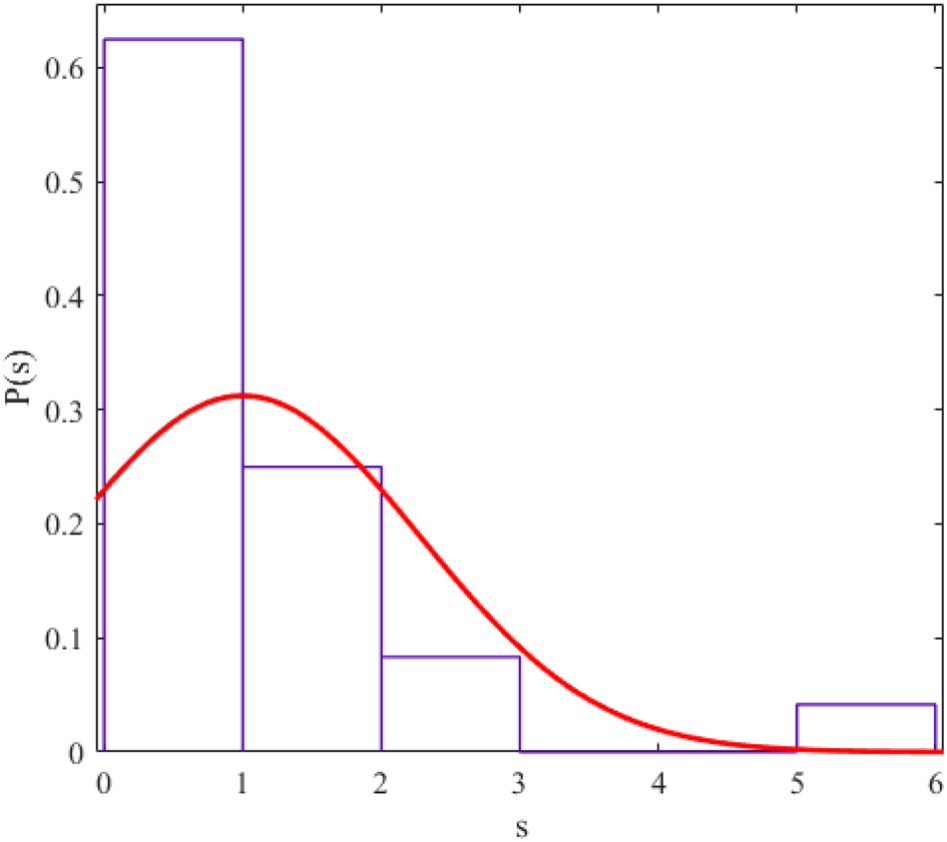
Similar to Fig. 1, the statistical distribution of $2_{\beta }^{ + }$ energy level.

**Figure 6:**
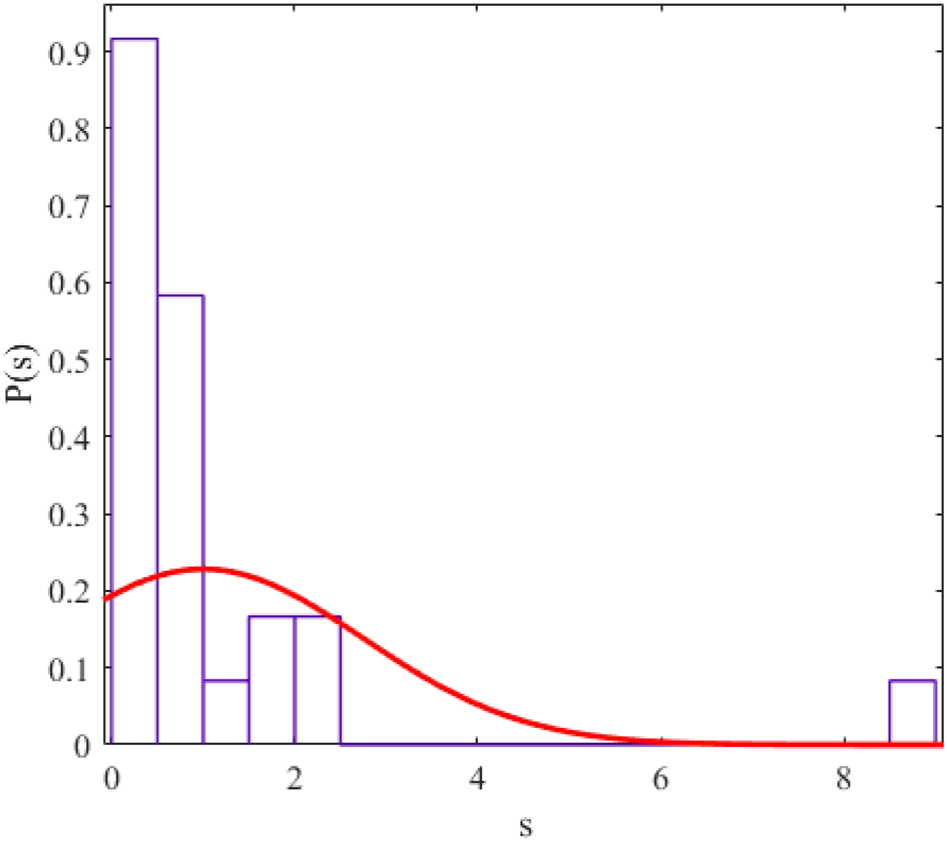
Similar to Fig. 1, the statistical distribution of $4_{\beta }^{ + }$ energy level.

**Figure 7:**
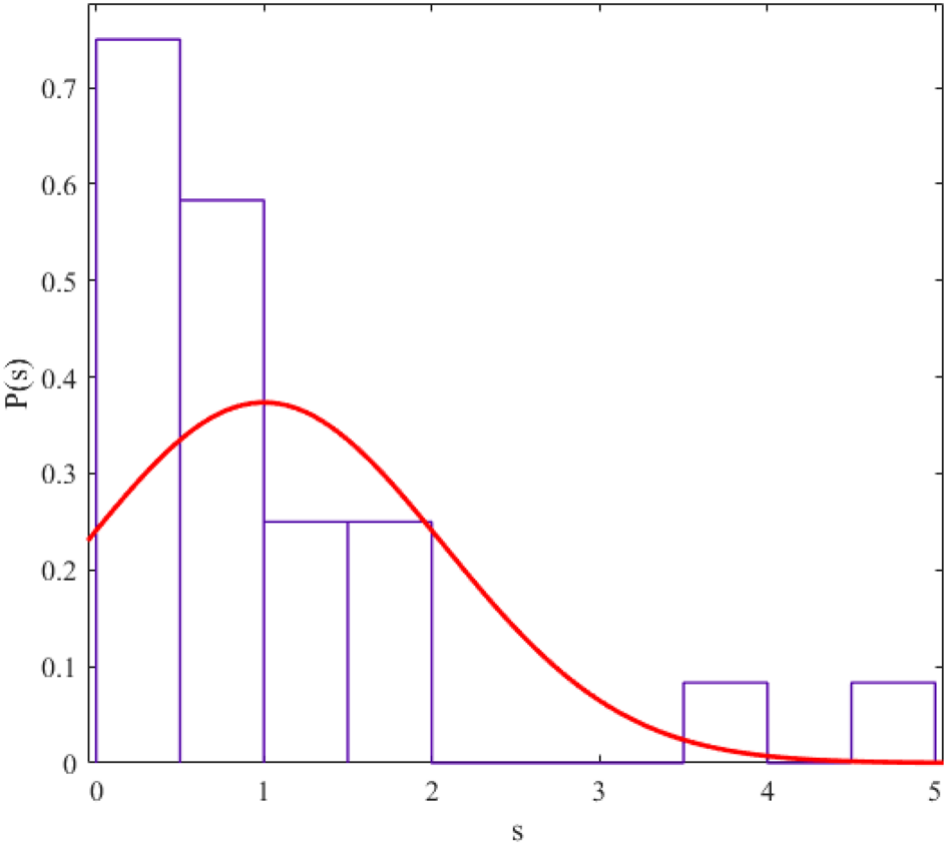
Similar to Fig. 1, the statistical distribution of $2_{{\upgamma }}^{ + }$ energy level.

**Figure 8:**
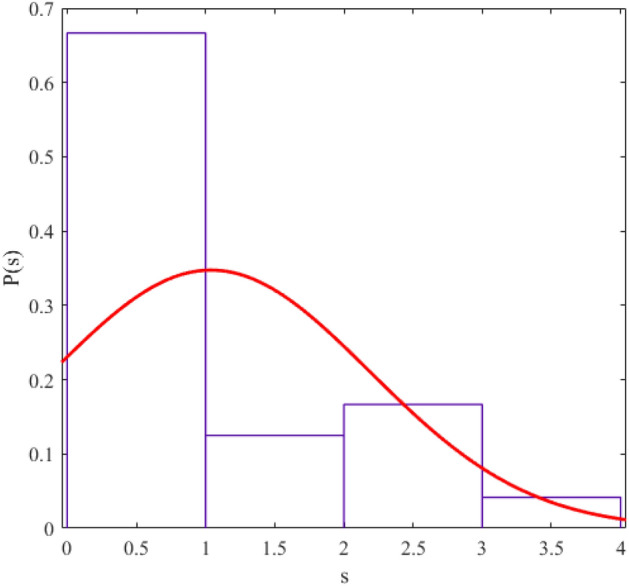
Similar to Fig. 1, the statistical distribution of $4_{{\upgamma }}^{ + }$ energy level.

The regularity and chaos of a system are determined through the similarity of the corresponding data distribution to the Gaussian distribution ($P\left( s \right) = \frac{\pi s}{2}\exp \left( {\frac{{\pi s^{2} }}{4}} \right)$) and Poisson distribution ($P\left( s \right) = e^{ - s}$). We fit a probability distribution function to the data to determine the degree of regularity in data distribution. The parameter value (obtained from the fitting process) allows us to give statistical labeling to our desired system. The probability distribution function we use in this article is the Berry-Robnik distribution (BRD) function^42–44^. The equation of the BRD function is in the form of

12 $$ P\left( s \right) = \left[ {q + \frac{\pi }{2}\left( {1 - q} \right)s} \right]\exp \left( { - qs - \frac{\pi }{4}\left( {1 - q} \right)s^{2} } \right) $$

where the parameter q = 0 represents the Gaussian distribution and q = 1 represents the Poisson distribution. We have shown the statistical distributions of our studied energy levels in Figs. 2, 3, 4, 5, 6, 7, 8.

Also, we presented q values (which we obtained from the curve fitting method in MATLAB software) in Table 4 for different levels of the regular nuclei.

**Table 4:** The values of the q parameter for different energy levels of the new regular nuclei candidates.

Energy levels	q
${\text{E}}(2_{{\text{g}}}^{ + } )$	0.2235
${\text{E}}(4_{{\text{g}}}^{ + } )$	0.1990
${\text{E}}(0_{{\upbeta }}^{ + } )$	0.1967
${\text{E}}(2_{{\upbeta }}^{ + } )$	0.0386
${\text{E}}(4_{{\upbeta }}^{ + } )$	0.1872
${\text{E}}(2_{{\upgamma }}^{ + } )$	0.1774
${\text{E}}(4_{{\upgamma }}^{ + } )$	0.0189

The results of the statistical study of energy levels (extracted from experimental sources) related to electromagnetic transitions indicate that the behavior of these levels in our considered nuclei is regular (their statistical distribution is similar to the Gaussian distribution). In other words, the reason for the presence of regularity in the "arc of regularity" region is due to the existence of structures similar to the nuclei in that area. Electromagnetic transition probabilities depend on the initial and final states. If the placement pattern of initial and final levels in the nuclear systems and their structure is similar, the probability ratio we defined also shows a similar pattern. Nuclei whose distance from the magic numbers for the number of neutrons is in the same range have almost the same energy spectrum structure. Hence, the obtained numbers for the ratios that we have defined for the electromagnetic transition rates exist for nuclei with a similar energy spectrum pattern empirically. For example, for ^104^Pd and ^108^Pd, 6 of the experimentally defined ratios do not exist. But most of the experimentally defined ratios have values for ^156^Gd and ^158^Gd (which are further away from the magic numbers). In Refs^45,46^, we also studied the role of the structure of nuclear systems and their distance from magic numbers in energy levels. On the other hand, the Electromagnetic transition probabilities depend completely to the wavefunctions of the initial and final levels which these quadrupole transitions are happened between them. This means, one can conclude that, the existence of such patterns in the energy spectra of the regular nuclei cause possible repetition schemes based on their quadrupole transitions. Also, this similarity between the energy spectra and the transition intensities in the framework of the IBM has reported in Ref^17^ by Karampagia, et al. for other nuclei.

## Conclusions

The regular nuclei were analyzed using a new measure based on their quadrupole transition rates. The results yielded by using all the available experimental data showed that these nuclei show a specific repetition pattern. Also, new candidates (for regular nuclei) were proposed by examining the existence of this behavior for all known isotopes. We also tested the location of these nuclei in the Casten triangle using the general IBM Hamiltonian, and the results of control parameters approved the situation of these 24 new suggested nuclei in the arc of regularity. The regularity concept in the definition of RMT is equivalent to the correlation of samples. We reported such analyses on the experimental energy spectra (related to electromagnetic transitions (of these nuclei. The regularity of the new nuclei was also confirmed by using the statistical study.

## Data Availability

The datasets used and analyzed during the current study available from the corresponding author on reasonable request.
